# Unknown Organofluorine Mixtures in U.S. Adult Serum:Contribution from Pharmaceuticals?

**DOI:** 10.3390/toxics11050416

**Published:** 2023-04-27

**Authors:** Emily H. Pennoyer, Wendy Heiger-Bernays, Rudolf Aro, Leo W. Y. Yeung, Jennifer J. Schlezinger, Thomas F. Webster

**Affiliations:** 1 Department of Environmental Health, Boston University School of Public Health, Boston, MA 02118, USA; 2 MTMResearch Centre, Örebro University, SE-701 82 Örebro, Sweden

**Keywords:** per- and polyfluoroalkyl substances, human biomonitoring, targeted analysis, extractable organofluorine, pharmaceuticals

## Abstract

Organofluorines occur in human serum as complex mixtures of known and unidentified compounds. Human biomonitoring traditionally uses targeted analysis to measure the presence of known and quantifiable per- and polyfluoroalkyl substances (PFAS) in serum, yet characterization of exposure to and quantification of PFAS are limited by the availability of methods and analytical standards. Studies comparing extractable organofluorine (EOF) in serum to measured PFAS using organofluorine mass balance show that measurable PFAS only explain a fraction of EOF in human serum and that other sources of organofluorine may exist. The gap in fluorine mass balance has important implications for human biomonitoring because the total body burden of PFAS cannot be characterized and the chemical species that make up unidentified EOF are unknown. Many highly prescribed pharmaceuticals contain organofluorine (e.g., Lipitor, Prozac) and are prescribed with dosing regimens designed to maintain a therapeutic range of concentrations in serum. Therefore, we hypothesize organofluorine pharmaceuticals contribute to EOF in serum. We use combustion ion chromatography to measure EOF in commercial serum from U.S. blood donors. Using fluorine mass balance, we assess differences in unexplained organofluorine (UOF) associated with pharmaceutical use and compare them with concentrations of organofluorine predicted based on the pharmacokinetic properties of each drug. Pharmacokinetic estimates of organofluorine attributable to pharmaceuticals ranged from 0.1 to 55.6 ng F/mL. Analysis of 44 target PFAS and EOF in samples of commercial serum (n = 20) shows the fraction of EOF not explained by Σ_44_ PFAS ranged from 15% to 86%. Self-reported use of organofluorine pharmaceuticals is associated with a 0.36 ng F/mL (95% CL: −1.26 to 1.97) increase in UOF, on average, compared to those who report not taking organofluorine pharmaceuticals. Our study is the first to assess sources of UOF in U.S. serum and examine whether organofluorine pharmaceuticals contribute to EOF. Discrepancies between pharmacokinetic estimates and EOF may be partly explained by differences in analytical measurements. Future analyses using EOF should consider multiple extraction methods to include cations and zwitterions. Whether organofluorine pharmaceuticals are classified as PFAS depends on the definition of PFAS.

## 1. Introduction

Per- and polyfluoroalkyl substances (PFAS) are a class of thousands of anthropogenic chemicals widely used in commercial products, industrial manufacturing, food packaging, pesticides, and aqueous film-forming foams for their durable and water-repellant properties [1]. PFAS are widely detected in the environment, in wildlife, and in humans [2,3,4,5,6,7]. A number of adverse health effects are associated with low levels of some PFAS in serum [8,9,10,11,12,13]. Traditional human biomonitoring studies measure 6–12 PFAS in serum [14], yet upwards of 12,000 individual PFAS are reported to exist [15]. The large number of PFAS makes a chemical-by-chemical approach to investigation unworkable, and class-based approaches to identify and track PFAS in humans and the environment based on their chemical structure have been proposed [16,17,18,19,20]. Further challenges are the differences in the definition of a PFAS [21]. Characterizing exposure to the full suite of PFAS is also limited by current analytical methods, which can only quantify exposure to compounds for which analytical standards exist and are readily available. Most of what is known about the health risks associated with PFAS comes from data on a very small subset of PFAS [22], yet less is known about the thousands of other PFAS in commerce [3].

Observations from the 1960s showed inorganic fluoride only partly explained the mass of total fluorine in serum, revealing unknown sources of organofluorine in serum [23]. In the 1990s and early 2000s, the identities of PFOA, PFOS, and other PFAS with industrial uses in the latter half of the 20th century were established [24], yet there remains a gap in our knowledge of the contributors to the fluorine detected in serum. Novel methods using combustion ion chromatography (CIC) for fluorine show large amounts of extractable organofluorine (EOF) in serum [25]. Using fluorine mass balance approaches, pioneering studies have shown that *conventional* PFAS—defined here as those with industrial uses and their precursors and replacements that are typically measured in human biomonitoring—only partly explain EOF in serum, with the fraction of unexplained organofluorine (UOF) ranging from 24% to 89% [25,26,27,28].

There are a number of potential types of UOF in human serum. (1) Replacements for conventional PFAS or their precursors, for which analytical standards are not available. For example, perfluoroalkyl acid (PFAA) precursors can be metabolized in humans to form terminal PFAS species [2], but the vast array of PFAS precursors as well as their respective intermediates are not quantifiable using traditional liquid chromatography mass spectrometry (LC-MS/MS) techniques [27,29,30,31]. (2) Ultra-short-chain PFAS that are not typically biomonitored. For example, trifluoroacetic acid (TFA) is a breakdown product of some organofluorine compounds that is frequently detected in the environment but only rarely measured in serum [27]. (3) Organofluorine pharmaceuticals. (4) Other organofluorine compounds, including pesticides. Whether these additional sources contribute to UOF as “PFAS” depends on the structural definition of PFAS being used.

Many highly prescribed pharmaceuticals contain organofluorine and can be classified as PFAS under some definitions [21]. Organofluorine has been used in the pharmaceutical industry since 1954 and is useful in altering the physiochemical properties of a drug to achieve a desired pharmacological effect [32]. Today, roughly 20% of pharmaceuticals currently registered with the FDA contain organofluorine [32], including the frequently prescribed anti-depressant Prozac, the protease inhibitor Paxlovid used for treating COVID-19, and Lipitor, the top-prescribed medication in the U.S. and used to lower cholesterol. With the correct dosing regimen, pharmaceuticals are prescribed in such a way that they remain present in serum within a clinically effective range [33].

We hypothesize that a portion of UOF in serum is attributable to organofluorine pharmaceuticals. In this pilot study using human serum, we used fluorine mass balance to assess differences in UOF associated with reported pharmaceutical use and compared them with concentrations of organofluorine predicted based on the pharmacokinetic properties of each pharmaceutical. To our knowledge, this is the first study to use CIC for fluorine to measure EOF in U.S. serum and determine the fraction of EOF not explained by conventional PFAS.

## 2. Materials and Methods

### 2.1. Organofluorine Pharmaceutical Selection

To identify highly prescribed organofluorine pharmaceuticals likely to be used among a random sample of U.S. adults, we used the list of 340 organofluorine pharmaceuticals approved in the U.S., Japan, and Europe between 1954 and 2019 generated by Inoue et al. [32] based on the publicly available KEGG drug database [34]. The KEGG Drug Database is a publicly available repository of approved drugs in the U.S., Europe, and Japan, which includes their chemical properties and molecular structures, as well as other identifiers for prescription and over-the-counter (OTC) pharmaceuticals. Cross-referencing the 340 organofluorine pharmaceuticals with drug utilization data from the ClinCalc Drug Database (version 2022.08) [35], we identified nine organofluorine pharmaceuticals that ranked among the top 100 prescribed drugs in the U.S. in 2020 (Table 1), therefore representing the organofluorine pharmaceuticals most likely to be used among U.S. adults.

### 2.2. Pharmacokinetic Estimates

We used the pharmacokinetic properties of the nine organofluorine pharmaceuticals shown in Table 1 to estimate the concentration of organofluorine in serum attributable to each of these compounds. Pharmaceuticals are prescribed to maintain blood concentrations within a therapeutic range, i.e., the steady state. The average concentration at steady state is commonly used to estimate levels of a compound in serum. For purposes of this analysis, we assumed steady-state concentrations for each of the nine pharmaceuticals were achieved given that all the compounds are prescribed for chronic conditions [21] with dosing regimens that aim to maintain relatively constant levels of the compound within a therapeutic range. Pharmacokinetic information was obtained from FDA drug labels when available and from the clinical literature when FDA drug labels were incomplete. For each pharmaceutical, the estimated range of serum levels of the parent compound (ng/mL) was represented by the range of average plasma concentrations at steady state (*C_SS_*). When available, we used the range of average plasma concentrations reported in the drug label or the literature. Otherwise, we used the pharmacokinetic parameters in Equation (1) to determine the average plasma concentration at steady state.

(1) $$C_{SS}=\frac{AUC}{\tau }$$

*C_SS_* is the average concentration at steady state (ng/mL), the area under the curve (*AUC*) is equivalent to the total exposure to the compound over the course of the dosing interval (ng*h/mL), and *τ* is the duration of the dosing interval in hours (h).

To determine the concentration of organofluorine in serum attributable to a given compound, we multiplied the proportion of the compound due to fluorine by the serum concentration of the compound (Equation (2)) [27].

(2) $$C_{F}=\left(\#\;\mathrm{flourine}\;\mathrm{atoms}\times \frac{MW_{F}}{MW_{C}}\right)\times C$$

where *C_F_* (ng F/mL) is the concentration of the compound in equivalents of fluorine, *MW_F_* is the atomic weight of fluorine (g/mol), *MW_C_* is the molecular weight of the compound (g/mol), and *C* is the concentration of the compound in serum (ng/mL), for pharmaceuticals the range of steady-state concentrations.

### 2.3. Serum Procurement and Sampling Protocol

All serum samples were sourced commercially from BioIVT Laboratory Services (Westbury, NY, USA), a biospecimen procurement company. Samples were collected from consenting donors by BioIVT at blood collection centers across the U.S. between 10 December 2020, and 18 January 2021 [41]. Per this study-specified sampling protocol, donors ages 25–65 with no previous history of taking cholestyramine (Questran), a cholesterol-lowering medication associated with increased elimination of PFAS [42], were eligible for inclusion in the study. At the time of sample collection, donors were verbally screened by BiolVT to ascertain their current medication use, and information was collected on donor demographics related to age, sex, and race/ethnicity.

We provided BioIVT with a list of the generic drug names and corresponding brand names for the nine organofluorine pharmaceuticals in Table 1. Serum was collected from ten males and ten females, half of whom reported using one or more of the nine organofluorine pharmaceuticals at the time of sampling, and the other half reported not using these pharmaceuticals. We classified the first group as pharmaceutical users and the second as non-users.

### 2.4. Sample Handling

As per BioIVT internal standard operating procedures, individuals’ whole blood was drawn into a 500 mL dry collection bag (Terumo BCT, INC., Lakewood, CO, USA, model 1BB*D606A) and spun at 5000× *g* for 10 min at −5 °C. The supernatant liquid was transferred into another bag and allowed to clot at room temperature for up to 48 h, and then spun at 5000× *g* for 20 min at −4 °C. The supernatant liquid was aliquoted into individual 3 mL vacutainers, frozen on dry ice for transport, and shipped via Boston University SPH to Örebro University, Örebro, Sweden, where it was stored at −20 °C and analyzed for PFAS and EOF.

### 2.5. Sample Extraction

The sample extraction method used was based on an ion-pair extraction method originally published by Hansen et al. [24] and later modified in this study. In brief, the samples were extracted in duplicate; the first subsample (Replicate 1) was used for target analysis (spiked with an internal standard), and the second subsample (Replicate 2) was used for EOF analysis (no internal standard added). First, 2 mL of a 0.5 M TBA solution in water was added to the sample and vortex mixed; then, 5 mL of MTBE was added to the mixture. The mixture was shaken horizontally at 250 rpm for 15 min and then centrifuged for 10 min at 8500 rpm (8000× *g*). The extraction was repeated twice with 3 mL of MTBE instead of 5 mL after collecting the organic solvent layer. The organic solvent extracts from all three cycles were combined and evaporated to 0.2 mL under a stream of nitrogen, then reconstituted to 1.0 mL with MeOH and evaporated to a final volume of 0.5 mL for instrumental analysis.

### 2.6. Instrumental Analysis and Quantification

The analysis and quantification have been described in detail previously [43]. In brief, the target analytes were quantified using an Acquity ultra-performance liquid chromatograph (UPLC) with a Xevo TQ-S micro tandem mass spectrometer (MS/MS). Both instruments were from the Waters Corporation (Milford, MA 01757, United States). The target analytes were separated using a C18 BEH column (2.1 × 100 mm, 1.7 μm); the mobile phases were a 30/70 (*v*/*v*) mixture of MeOH and water and 100% MeOH. Both mobile phases had 5 mmol/L 1-methylpiperidine and 2 mmol/L ammonium acetate as additives [44]. The 44 target PFAS included legacy compounds, potential precursors, and novel PFAS species, which were quantified using internal calibration with corresponding internal standards (mass-labelled standards). For those without a corresponding internal standard, the compound with the closest retention time of the same compound class was used. Details of the list of internal standards used can be found elsewhere [27]. Repeated injections of a standard mixture during analytical runs were used to monitor the performance of the UPLC-MS/MS system.

A combustion ion chromatography (CIC) system was used to determine EOF content. The CIC system was made of a combustion module (Analytik Jena, Germany), a 920 absorber module (Metrohm, Switzerland), and a 930 compact IC flex ion chromatograph module (Metrohm, Switzerland). EOF analysis results are described in ng F/g, which are equivalent to the concentration of fluorine in serum (ng F/mL) using the density of serum (~1 g/mL). Separation was achieved using an ion-exchange column (Metrosep A Supp 5–150/4.0), and the mobile phase was a carbonate buffer (64 mmol/L sodium carbonate and 20 mmol/L sodium bicarbonate). Water was used as the absorber solution. An external calibration curve, produced by combusting PFOA (Aldrich, Burlington, United States), was used for quantification.

The quality assurance and quality control (QA/QC) measures, including the performance of samples using SRM1957 and the relative standard deviation (RSD) of QC samples consisting of PFOS and PFOA, recoveries of target PFAS, as well as repeatability of the CIC system, are described in more detail elsewhere [43]. The limits of quantification (LOQ) of individual PFAS ranged from 0.020 to 0.065 ng/mL (Supplemental Information Table S1). To calculate ΣPFAS values, measurements of target PFAS below the LOQ were substituted with the value of zero, as described elsewhere [43]. The LOQ (3.8 ng F/g) of EOF using CIC was calculated as the average of three procedural blanks plus three times the standard deviation of the procedural blanks before applying any concentration factor for the volume of blood used. Samples with values below the LOQ were replaced with the LOQ value divided by the concentration factor of the sample. Reported sample concentrations might result in lower values than the LOQ value due to different concentration factors (SI Table S2).

### 2.7. Organofluorine Mass Balance

Individual PFAS concentrations were converted to fluorine-equivalent concentrations using Equation (2), where C is now the serum concentration of the PFAS determined using LC-MS/MS. The known EOF for the measured 44 PFAS (Σ C_F-PFAS_; ng F/mL) was determined by summing the fluorine concentrations attributable to Σ_44_ PFAS. The concentration of unknown organofluorine (C_UOF_; ng F/mL) was calculated as the difference between the concentration of total EOF (C_EOF_; ng F/mL) and known EOF (Σ C_F-PFAS;_ ng F/mL).

### 2.8. Statistical Analysis

We used the Shapiro–Wilks test to assess the normality of the concentration of UOF. Since the distribution of UOF concentration was approximately normal, bivariate analyses were conducted using two sample t-tests to assess differences in UOF across sex, race/ethnicity, and age (treated as a dichotomous variable with observations falling above or below the median of 48.5 years). We used Spearman’s rank correlation to determine whether the concentration of UOF is associated with the concentration of organofluorine attributable to target PFAS.

We used linear regression to determine the crude difference in the concentration of UOF between donors who reported use of organofluorine pharmaceuticals and those who did not. Based on the results of bivariate analyses, we used multiple linear regression to consider the effect of age as a potential confounder in our estimates of the effect of pharmaceutical use on UOF. We performed regression diagnostics to confirm the assumptions of homoscedasticity, linearity, and independence were met and to identify potential influence points and outliers.

Given the skewed distribution of PFAS measured in both groups, we used the Wilcoxon rank-sum test to compare the distributions of Σ_44_ PFAS between pharmaceutical users and donors who reported no pharmaceutical use. To compare our sample with national levels reported by the National Health and Nutrition Examination Survey (NHANES), we compared the median concentrations of a subset of Σ_5_ PFAS monitored by NHANES for U.S. adults in 2017–2018 and detected in >95% of donated sera (n = 20): PFOA, PFOS, PFHxS, PFHpS, and PFNA (SI Table S2) [45].

## 3. Results

### 3.1. Pharmacokinetic Estimates of Serum Fluorine

Estimated serum organofluorine concentrations for each of the nine pharmaceuticals are presented in Table 1. Concentrations of organofluorine attributable to each pharmaceutical range from 0.1 to 55.6 ng F/mL, directly influenced by the number of fluorine atoms in their molecular structure. Atorvastatin (Lipitor), estimated to contribute 0.1–0.4 ng F/mL, is the most widely used drug of the nine, with over 114 million U.S. prescriptions per year in 2020. On the high end, Fluoxetine (Prozac) contributes 15.8–55.6 ng F/mL with 23 million prescriptions per year. Two compounds, Citalopram and Escitalopram, share the same molecular formula, but the former is a mixture of two enantiomers while the latter is one enantiomer and is prescribed at half the dose, explaining why the estimated levels of organofluorine in serum differ by a factor of two.

### 3.2. Characteristics of Study Serum Donors and PFAS Concentrations

Demographic data for the serum donor population are presented in Table 2, PFAS results are presented in Table 3, and Supplemental Information is presented in Table S2. Donors that reported using pharmaceuticals were on average five years older than donors with no reported pharmaceutical use but did not differ in their median age (Table 2). Pharmaceutical users had somewhat higher serum concentrations of Σ_44_ PFAS (Table 3).

The median values of Σ_5_ PFAS (SI Table S2) were lower in female donors compared to male donors, which is consistent with data from NHANES (data not shown) [45]. For both males and females in our study, median concentrations of Σ_5_ PFAS were lower than national levels reported in NHANES in 2017–2018; however, 100% of donors in our study identified as Black or Hispanic, who have lower median levels of PFAS compared to non-Hispanic white populations in NHANES [45,46].

### 3.3. Extractable Organofluorine in Serum

Concentrations of EOF observed in our study ranged from <2.02 to 11.2 ng F/mL and were slightly higher amongst the pharmaceutical users (Table 3). Consistent with NHANES, the individual PFAS analytes comprising the majority of identified EOF were linear and branched PFOS, collectively accounting for roughly 50%, followed by PFHxS (23%), and PFOA (14%) (SI Table S2). The concentration of EOF was similar across Black and Hispanic donors (data not shown).

### 3.4. Unexplained Organofluorine in Serum

**Figure 1:**
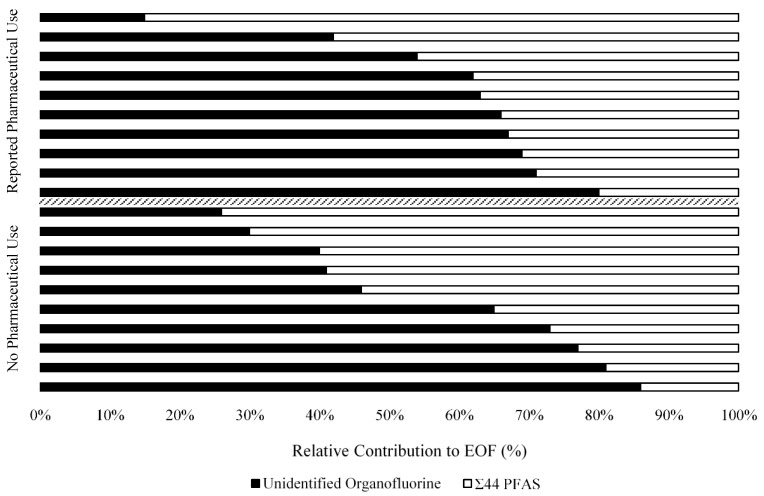
Relative contribution of unidentified organofluorine and Σ_44_ PFAS to extractable organofluorine (EOF) (%) in individual serum samples from donors who report using select organofluorine pharmaceuticals (n = 10) and those who do not (n = 10).

The proportion of UOF relative to EOF measured in serum ranged from 15% to 86% (Figure 1), which is comparable with previous studies that show the proportion of UOF ranging from 30% to 70% [26,27]. The distribution of UOF (ng F/mL) in our study was approximately normal and ranged from 0.94 to 7.48 ng F/mL (Table 3). The mean concentration of UOF was slightly lower in serum from Black donors compared to Hispanic donors and slightly greater in females compared to males, but neither difference was statistically significant (*p* > 0.05) (Table S3a,b). On average, study participants above the median age of 48.5 had a 1.4 ng F/mL greater concentration of UOF than those below the median age (*p* = 0.056) (Table S3c). The concentration of UOF and fluorine attributable to Σ_44_ PFAS do not appear to be correlated, with a Spearman correlation coefficient of ρ = 0.06 (*p*-value = 0.82) suggesting that contributors to UOF are not associated with the fluorine attributed to the 44 PFAS measured in serum.

### 3.5. Linear Regression of UOF on Pharmaceutical Use

**Figure 2:**
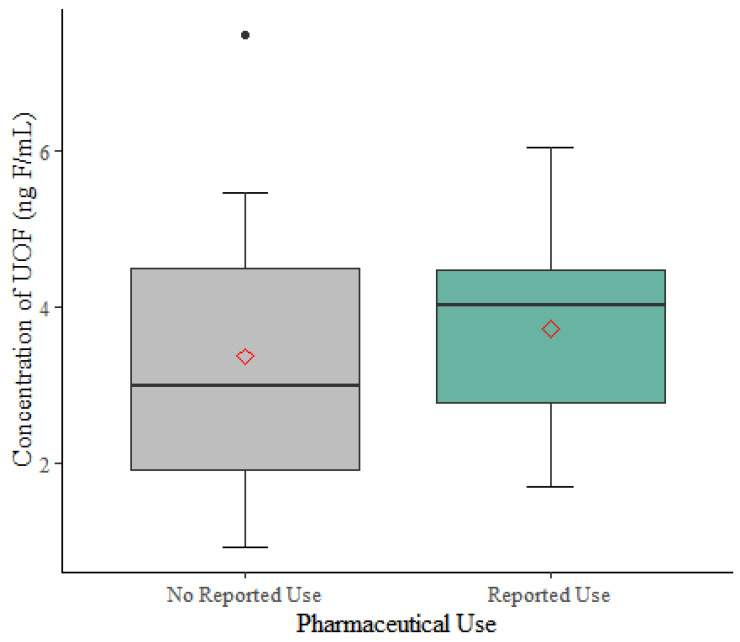
Boxplots of UOF measured in donated serum (n = 20). Whiskers range from 1 SD above and below the mean for each group with the mean and median observation for UOF among reported users of nine organofluorine pharmaceuticals and non-users.

Comparing the difference in the concentration of UOF between groups, people who report using organofluorine pharmaceuticals had 0.36 ng F/mL greater UOF, on average, compared to people who reported not using these pharmaceuticals (95% CI: −1.26, 1.96, Figure 2), but the difference was not statistically significant at the α = 0.05 level. Adjusting for age had no effect on the relationship between pharmaceutical use and the concentration of UOF (Table 4). Diagnostic tests showed the linear model did not violate regression assumptions. We identified one potential outlier; omitting the observation, the crude mean difference in UOF between pharmaceutical users and non-users increased to 0.81 ng F/mL (95% CI: −0.56 to 2.18).

## 4. Discussion

Previous studies using organofluorine mass balance revealed the occurrence of UOF in environmental and biological matrices [25,47,48,49,50,51], yet the characterization of total and unknown EOF in U.S. serum is not understood. In this study, we show that the concentration of EOF in serum from a sample of U.S. adults is only partially explained by conventional PFAS. The 44 PFAS we targeted account for 14–85% of EOF in serum, comparable with previous findings from China, which showed the concentration of Σ_10_ PFAS accounted for 30–70% of EOF [26], and from Sweden, which showed the concentration of Σ_61_ PFAS accounted for 30–74% of EOF [27]. Substituting zero for left-censored values used to calculate Σ_44_ PFAS in our study may underestimate the fraction of EOF explained by targeted PFAS.

Previous studies in Sweden suggest UOF may differ by sex and age [27]. Bivariate analyses in our study suggested a small difference by sex (UOF slightly increased in females) and a larger difference by age (higher above the median age than below). Age did not appear to confound the relationship between UOF and reported use of organofluorine pharmaceuticals, but the small sample size in this exploratory study limited further examination of possible confounders. Importantly, limited information on the commercial donor population and demographics besides sex, age, and race/ethnicity reduces our ability to generalize results to other populations.

Our results suggest people who reported using organofluorine pharmaceuticals have a slightly greater concentration of UOF (0.36 ng F/mL) compared to those who do not report using these pharmaceuticals. While this difference is consistent with the estimated organofluorine concentrations contributed by some drugs (e.g., Lipitor and Crestor), it is two orders of magnitude lower than some others (Table 1). If taken as prescribed, organofluorine pharmaceuticals should exist in serum at relatively stable levels, and the estimated concentration of organofluorine attributable to some pharmaceutical compounds exceeded 40 ng F/mL (i.e., Prozac, Januvia). For comparison, the median blood level for PFOS in the general U.S. population in 2017–2018 was 4.30 ng/mL and 1.47 ng/mL for PFOA [45].

There are at least two possible explanations for the discrepancy between the pharmacokinetic estimates and the analysis of EOF in serum: (1) uncertainties in knowledge about pharmaceutical use; and (2) analytical approaches to the quantification of EOF related to pharmaceuticals in serum. We assumed that the pharmaceuticals were in steady state, using the average concentration at steady state to represent the range of levels that would be expected upon continuous administration of a drug, yet we lacked information on the duration, frequency, or compliance of serum donors for the pharmaceuticals they reported using. We also lacked information on socioeconomic status that could influence whether pharmaceuticals are used as prescribed in this population (e.g., adherence) and whether the results can be generalized to other populations. Self-reported pharmaceutical use could introduce non-differential misclassification of exposure if donors did not accurately recall the names of their medications or if they did not truthfully report their medication use (e.g., because of associated social stigma [52]). This misclassification would bias our results towards the null. Furthermore, people may not take the pharmaceuticals as prescribed (e.g., accidentally or intentionally skipping doses), though the slow elimination rates of some organofluorine pharmaceuticals make it likely for the compound to persist in the body for days to weeks even if dosing is skipped or stopped [53].

Discrepancies between the pharmacokinetic estimates and the EOF analysis may also be explained by differences in analytical measurements. We used ion-pair extraction, a method shown to capture some PFAS (neutral, sulfonates, and carboxylates); however, the capability for capturing cationic or zwitterionic compounds varies and depends on chain length [54]. Depending on the functional groups and the dissociation constant, organofluorine pharmaceuticals can be neutral, anionic, cationic, or zwitterionic at physiological pH (Table 1), as can some “PFAS” [1,55]. Since no alkaline buffer was used for the ion-pair extraction, Januvia, Prozac, Citalopram/Escitalopram, and Paxil (Table 1), each of which exist as cations at physiologic pH, may not be captured using conventional extraction methods developed for anionic compounds. It is possible that traditional extraction techniques for anionic compounds do not capture the full suite of organofluorine compounds in a sample, and true EOF is likely much larger, particularly in samples where cationic organofluorine species are present. Furthermore, our analysis was limited to pharmacokinetic estimates for organofluorine from parent compounds, not considering the contributions from fluorinated metabolites that can also accumulate in serum. For example, fluoxetine (Prozac) is extensively metabolized into norfluoxetine, which is measured at concentrations of 72–258 ng F/mL and has a fluorine equivalent of 13–47 ng F/mL [53]. Fluorinated metabolites exist for other organofluorine pharmaceuticals as well, but differences in pharmacokinetics related to age, sex, diet, genetic polymorphisms in metabolizing enzymes, and drug-drug interactions make estimating the organofluorine contribution from active and inactive metabolites more complicated [56]. Therefore, the estimated concentration of organofluorine in serum attributable to pharmaceuticals is likely even greater, with true EOF accounting for contributions from organofluorine pharmaceuticals and metabolites.

Our results suggest that organofluorine pharmaceuticals contribute to EOF, but that a substantial amount of EOF remains unexplained. Large fractions of UOF among people who report not using the nine organofluorine pharmaceuticals suggest other sources of UOF. Other sources of EOF not measured in this study may include pesticides, ultra-short-chain organofluorine compounds such as TFA, as well as PFAS or their precursors, for which analytical standards are not available or have not yet been identified. We did not analyze ultra-short-chain PFAS in our study, though one study in Sweden detected TFA in >60% of blood samples [27]. While short-chain compounds typically have shorter biological half-lives [57], continuous exposure to these compounds in the environment may contribute to EOF.

Recent studies using EOF as a class-based analytical method to screen for PFAS in environmental media may wish to understand the extent to which unknown PFAS contribute to contamination [58]. However, whether organofluorine compounds such as TFA or pharmaceuticals contribute to EOF as “PFAS” depends on the definition of PFAS being used and the user-specific working scope. For example, as written, the definition developed by the U.S. Department of Defense for the purpose of monitoring for PFAS in surface waters includes 94% of organofluorine pharmaceuticals [21]. In this context, measuring the presence of pharmaceuticals could be of great importance, and analyses using EOF to screen for PFAS should consider using multiple extraction methods that can measure anions, cations, and zwitterions because organofluorine pharmaceuticals are present in surface water [21,59]. Non-pharmaceutical organofluorines also exist as cations and zwitterions [1,55]. All of these compounds would contribute to EOF if fully extracted, yet whether they contribute as “PFAS” depends on how PFAS are defined and the context in which they are studied. Future analyses using EOF to screen for PFAS may consider multiple extraction methods to detect these compounds in environmental and biological media.

## 5. Conclusions

Since the detection of organofluorine in serum in the 1960s, efforts to close the fluorine mass balance gap rely on adequate analytical methods and standards to identify, detect, and quantify compounds of interest. Here, we present an illustrative example highlighting the importance of using appropriate analytical methods for the context of the analysis. The definition of PFAS has important implications for organofluorine mass balance, as the fraction of EOF explained by “PFAS” depends on the definition being used. Depending on the purpose for which a definition is being used (e.g., water quality monitoring, regulatory action to ban PFAS in consumer products) [21], the implications for EOF and the inclusion of cations and zwitterions may vary. Our findings suggest organofluorine pharmaceuticals contribute to EOF in serum, but a large fraction of EOF remains unexplained. Future analyses should consider multiple extraction methods to also include cations and zwitterions.

## Figures and Tables

**Table 1:** Top nine U.S. prescribed organofluorine pharmaceuticals, names, uses, chemical properties, and pharmacokinetic-based estimates of organofluorine in order of U.S. prescriptions (2020).

Pharmaceutical Information				Chemical Properties						
Generic Name	Brand Name	Therapeutic Use	U.S. Prescriptions (2020)	Molecular Formula	Molecular Weight (g/mol)	Dissociation Constant (pka) ^a^	#F	Fraction F	Estimated Serum Levels (ng/mL) ^b^	Estimated Organofluorine (ng F/mL)
Atorvastatin	Lipitor	statin	114,509,814	C_33_H_35_FN_2_O_5_	558.65	4.31	1	0.034	2.4–11.2 ^d^	0.1–0.4
Escitalopram	Lexapro	SSRI	30,605,646	C_20_H_21_FN_2_O	324.40	9.78	1	0.059	15–80 ^e^	0.9–4.7
Rosuvastatin	Crestor	Statin	29,750,488	C_22_H_28_FN_3_O_6_S	481.54	4.00	1	0.039	1.8–7.4 ^f, g^	0.1–0.3
Pantoprazole	Protonix	Proton-pump inhibitor	26,604,040	C_16_H_15_F_2_N_3_O_4_S	383.37	3.92 (SB)8.19 (SA)	2	0.099	39.9–87.9	4.0–8.7
Fluticasone ^c^	Flonase	intranasal corticosteroid	24,777,490	C_22_H_27_F_3_O_4_S	444.51	−3.4 (SB)13.56 (SA)	3	0.128	N/A	N/A
Fluoxetine	Prozac	SSRI	23,403,050	C_17_H_18_F_3_NO	309.33	9.80	3	0.184	91–302	15.8–55.6
Citalopram	Celexa	SSRI	18,549,176	C_20_H_21_FN_2_O	324.40	9.78	1	0.059	50–100 ^e^	2.9–6.4
Sitagliptin	Januvia	anti-diabetic	9,885,657	C_16_H_18_F_6_N_5_O_5_P	407.32	8.78	6	0.280	165	46.3
Paroxetine	Paxil	SSRI	9,029,667	C_19_H_20_FNO_3_	329.37	9.77	1	0.058	30–120 ^e^	1.7–6.9

Notes: ^a^ pKa values obtained from DrugBank Database [36].; ^b^ All pharmacokinetic data obtained from FDA drug labels unless otherwise noted.; ^c^ Limited pharmacokinetic information available for oral inhalation of Fluticasone.; ^d^ Lins et al., 2003 [37]; ^e^ Mayo Clinic Labs Test Catalog [38]; ^f^ Li et al., 2010 [39]; ^g^ Martin et al., 2002 [40]; Abbreviations: SSRI: selective serotonin reuptake inhibitor; Pantoprazole and Fluticasone are zwitterions with multiple functional groups; pKa values are provided for each functional group, denoted as strongest acid (SA) and strongest base (SB).

**Table 2:** Demographic data for blood donors (n = 20). Donors who reported a history of taking Questran were excluded.

	No Reported Pharma Use (n = 10)	Reported Pharma Use (n = 10)
	n (%)	n (%)
**Sex**		
Male	5(50)	5(50)
Female	5(50)	5(50)
**Race**		
Black	4(40)	4(40)
Hispanic	6(60)	6(60)
**Age**		
mean (±SD)	45 (±11.8)	50 (±13.4)
median	49	48.5
range	28–59	32–74

**Table 3:** Distribution of Σ_44_PFAS, EOF and UOF among donors who report using select organofluorine pharmaceuticals and those who do not.

	No Reported Pharma Use (n = 10)	Reported Pharma Use (n = 10)
**Concentration of Σ_44_ PFAS** (ng/mL)		
mean (±SD)	6.54 (±3.55)	9.51 (±7.35)
median	5.87	7.49
range	3.16–14.90	2.88–26.24
**Concentration of EOF** (ng F/mL) ^a^		
mean (±SD)	6.10 (±2.59)	6.93 (±2.76)
median	6.45	6.26
range	2.02–10.04	2.67–11.22
**Concentration of UOF** (ng F/mL) ^b^		
mean (±SD)	3.37 (±2.04)	3.73 (±1.31)
median	2.99	4.02
range	0.94–7.48	1.70–6.05

Notes: ^a^ EOF was measured using CIC for fluorine.; ^b^ UOF was determined as the concentration of EOF not explained by fluorine attributable to Σ_44_ PFAS.

**Table 4:** Linear regression estimating the relationship between unexplained organofluorine (ng F/mL) and reported pharmaceutical use, adjusting for age.

Variable	Coefficients (95% CI)	Standard Error
Intercept	2.65 (1.36 to 3.94)	0.61
Organofluorine Pharmaceutical Use	0.36 (−1.14 to 1.85)	0.71
Age ^a^	1.43 (−0.06 to 2.93)	0.71

Notes: ^a^ Model is adjusted for age (above or below median age of 48.5).

## Data Availability

Research data can be found in the Supplemental Information.

## Supplementary Materials

The following supporting information can be downloaded at: https://www.mdpi.com/article/10.3390/toxics11050416/s1. Table S1: Analytes for targeted LC-MS/MS and their limits of quantification (LOQ); Table S2: Concentrations of target PFAS per subject (ng/mL), extractable organofluorine (EOF; ng F/mL), and the concentration of fluorine attributable to total PFAS (F44-PFAS; ng F/mL); Tables S3: Unexplained organofluorine (UOF) stratified by sex, race, and age.
