# Supplementary material for: Unknown Organofluorine Mixtures in U.S. Adult Serum:Contribution from Pharmaceuticals?
Source: Toxics. 2023 Apr 27;11(5):416. doi: 10.3390/toxics11050416 (PMC10221009; doi:10.3390/toxics11050416)
Supplement: Supplementary file 1 [file toxics-11-00416-s001.zip › toxics-2316311-supplementary.zip!/toxics-2316311-supplementary.pdf]

SUPPLEMENTAL INFORMATION:

Table S1. Analytes for Targeted LC-MS/MS and their limits of quantification (LOQ).

**Name**	**Acronym**	**Classification**	**Limit of Quantification (ng/mL)**
Perfluorobutanoate	PFBA	Short-chain	0.05
Perfluoropentanoate	PFPeA	Short-chain	0.02
Perfluorohexanoate	PFHxA	Short-chain	0.05
Perfluoroheptanoate	PFHpA	Short-chain	0.04
Perfluorooctanoate	PFOA	Long-chain	0.02
Perfluorononanoate	PFNA	Long-chain	0.02
Perfluorodecanoate	PFDA	Long-chain	0.065
Perfluoroundecanoate	PFUnDA	Long-chain	0.02
Perfluorododecanoate	PFDoDA	Long-chain	0.02
Perfluorotridecanoate	PFTrDA	Long-chain	0.02
Perfluorotetradecanoate	PFTDA	Long-chain	0.02
Perfluorohexadecanoic acid	PFHxDA	Long-chain	0.05
Perfluorooctadecanoic acid	PFOcDA	Long-chain	0.05
Perfluorobutane sulfonate	PFBS	Short-chain	0.02
Perfluoropentane sulfonate	PFPeS	Short-chain	0.02
Perfluorohexane sulfonate	PFHxS	Long-chain	0.03
Perfluoroheptane sulfonate	PFHpS	Long-chain	0.02
Perfluorooctane sulfonate	PFOS	Long-chain	0.04
m2-perfluorooctane sulfonate	Dimethyl-PFOS	Long-chain	0.02
3,4,5-m-perfluorooctane sulfonate	3/4/5-m-PFOS	Long-chain	0.04
6, 2-m-perfluorooctane sulfonate	6/2-m-PFOS	Long-chain	0.05
Perfluoro-1-methylheptane sulfonate	1-m-PFOS	Long-chain	0.02
Perfluorononane sulfonate	PFNS	Long-chain	0.02
Perfluorodecane sulfonate	PFDS	Long-chain	0.02
Perfluorododecane sulfonate	PFDoDS	Long-chain	0.02

Perfluorobutane sulfonamide	FBSA	PFSA precursor	0.04
Perfluorohexane sulfonamide	PFHxSA	PFSA precursor	0.04
N-Methyl perfluorohexane sulfonamide	Me-PFHxSA	PFSA precursor	0.02
Perfluorooctane sulfonamide	FOSA	PFSA precursor	0.05
3:3 fluorotelomer carboxylic acid	3:3 FTCA	PFCA precursor	0.05
5:3 fluorotelomer carboxylic acid	5:3 FTCA	PFCA precursor	0.02
7:3 fluorotelomer carboxylic acid	7:3 FTCA	PFCA precursor	0.05
6:2 fluorotelomer carboxylic acid	6:2 FTUCA	PFCA precursor	0.03
8:2 fluorotelomer carboxylic acid	8:2 FTUCA	PFCA precursor	0.03
10:2 fluorotelomer carboxylic acid	10:2 FTUCA	PFCA precursor	0.03
4:2 fluorotelomer sulfonate	4:2 FTSA	PFCA precursor	0.02
6:2 fluorotelomer sulfonate	6:2 FTSA	PFCA precursor	0.02
8:2 fluorotelomer sulfonate	8:2 FTSA	PFCA precursor	0.02
10:2 fluorotelomer sulfonate	10:2 FTSA	PFCA precursor	0.02
Perfluorohexylphosphonic acid	PFHxPA	Potential precursor	0.05
C6/C6 perfluorophosphinic acid	C6/C6 PFPiA	Potential precursor	0.05
C6/C8 perfluorophosphinic acid	C6/C8 PFPiA	Potential precursor	0.05
C8/C8 perfluorophosphinic acid	C8/C8 PFPiA	Potential precursor	0.05
2,3,3,3-tetrafluoro-2-(heptafluoropropoxy)propanoate	HFPO-DA (GenX)	Novel species	0.02
4,8-dioxa-3H-perfluorononanoic acid	ADONA	Novel species	0.02
Perfluoroethylcyclohexane Sulfonate	PFECHS	Novel species	0.02
6:2 chlorinated polyfluoroalkyl ether sulfonic acid	6:2 Cl-PFESA	Novel species	0.02
8:2 chlorinated polyfluoroalkyl ether sulfonic acid	8:2 Cl-PFESA	Novel species	0.02

Table S2. Concentrations of target PFAS per subject (ng/mL), extractable organofluorine (EOF; ng/mL), and the concentration of fluorine attributable to total PFAS (F_{44PFAS}; ng F/mL)[1]

	Sex	Age	Σ_{44}PFAS[2]	Σ_{5}PFAS[3]	EOF[4]	F_{44PFAS}	PFOS[5]	PFHxS	PFNA	PFOA	PFHpS	PFECHS	PFBS	PFUnDA	PFHpA	PFPeS	PFTrDA	PFDoDA	FOSA	6:2 FTSA	PFPeA	PFDA	FBSA	6:2 Cl-PFESA
Reported Pharmaceutical Use	Male	50	4.99	4.71	7.36	2.79	2.87	0.80	0.31	0.66	0.07	0.26	0.02	<LOD	<LOD	<LOD	<LOD	<LOD	<LOD	<LOD	<LOD	<LOD	<LOD	<LOD
	Male	32	9.76	9.50	6.34	2.14	5.48	1.67	0.39	1.73	0.24	0.24	0.01	<LOD	<LOD	<LOD	<LOD	<LOD	<LOD	<LOD	<LOD	<LOD	<LOD	<LOD
	Male	47	9.86	8.68	6.08	2.77	5.01	1.78	0.54	1.26	0.09	0.72	0.08	0.07	0.24	0.02	<LOD	<LOD	<LOD	0.02	<LOD	<LOD	0.04	<LOD
	Male	65	26.24	24.64	11.22	9.52	14.44	7.07	0.90	1.81	0.42	1.54	0.03	0.02	<LOD	0.01	<LOD	<LOD	<LOD	<LOD	<LOD	<LOD	<LOD	<LOD
	Male	36	17.21	15.93	10.92	6.28	7.00	4.90	0.91	2.83	0.29	0.99	0.04	0.03	0.20	0.02	<LOD	<LOD	<LOD	<LOD	<LOD	<LOD	<LOD	<LOD
	Female	74	5.22	4.67	8.72	2.67	2.47	0.92	0.35	0.86	0.06	0.19	0.02	0.05	<LOD	<LOD	<LOD	<LOD	0.01	<LOD	0.28	<LOD	<LOD	<LOD
	Female	49	4.66	4.20	6.18	2.04	2.23	0.96	0.25	0.72	0.04	0.42	0.04	<LOD	<LOD	<LOD	<LOD	<LOD	<LOD	<LOD	<LOD	<LOD	<LOD	<LOD
	Female	48	11.01	9.93	6.18	2.28	6.68	1.05	0.81	1.29	0.11	0.21	0.02	0.11	<LOD	<LOD	<LOD	<LOD	<LOD	<LOD	<LOD	0.74	<LOD	<LOD
	Female	38	2.88	2.72	**3.66**	1.05	1.52	0.43	0.24	0.47	0.06	0.08	0.03	0.05	<LOD	<LOD	<LOD	<LOD	<LOD	<LOD	<LOD	<LOD	<LOD	<LOD
	Female	62	3.25	3.09	**2.67**	0.53	1.61	0.70	0.27	0.47	0.05	0.12	0.04	<LOD	<LOD	<LOD	<LOD	<LOD	<LOD	<LOD	<LOD	<LOD	<LOD	<LOD
No Pharmaceutical Use	Male	37	6.64	6.40	4.52	1.59	3.14	2.22	0.30	0.68	0.06	0.17	<LOD	0.06	<LOD	<LOD	0.01	<LOD	<LOD	<LOD	<LOD	<LOD	<LOD	<LOD
	Male	30	5.68	5.47	6.07	3.27	2.53	1.61	0.37	0.87	0.09	0.14	0.03	0.04	<LOD	<LOD	<LOD	<LOD	<LOD	<LOD	<LOD	<LOD	<LOD	<LOD
	Male	59	14.90	13.88	10.04	6.99	8.46	2.81	0.59	1.77	0.24	0.63	0.05	0.12	0.15	0.02	0.03	0.02	<LOD	<LOD	<LOD	<LOD	<LOD	<LOD
	Male	46	8.72	7.83	5.30	3.95	3.98	1.90	0.45	1.38	0.12	0.37	0.07	0.05	0.37	<LOD	<LOD	<LOD	<LOD	0.01	<LOD	<LOD	<LOD	0.02
	Male	52	6.06	5.87	6.84	1.86	3.28	0.97	0.60	0.93	0.09	0.15	0.04	<LOD	<LOD	<LOD	<LOD	<LOD	<LOD	<LOD	<LOD	<LOD	<LOD	<LOD
	Female	28	3.95	3.87	**2.02**	0.38	0.57	3.14	0.15	<LOD	<LOD	0.01	<LOD	0.05	<LOD	<LOD	<LOD	0.02	<LOD	<LOD	<LOD	<LOD	<LOD	<LOD
	Female	57	8.61	8.49	7.74	4.66	5.23	1.45	0.42	1.26	0.13	0.07	0.02	0.03	<LOD	<LOD	<LOD	<LOD	<LOD	<LOD	<LOD	<LOD	<LOD	<LOD
	Female	55	3.16	3.05	7.09	1.63	1.64	0.55	0.25	0.56	0.05	0.03	0.01	0.05	<LOD	<LOD	0.01	<LOD	<LOD	<LOD	<LOD	<LOD	<LOD	<LOD
	Female	33	3.16	3.16	**2.31**	1.37	1.80	0.47	0.27	0.57	0.05	<LOD	<LOD	<LOD	<LOD	<LOD	<LOD	<LOD	<LOD	<LOD	<LOD	<LOD	<LOD	<LOD
	Female	52	4.55	4.46	8.67	1.19	2.58	0.71	0.34	0.77	0.07	0.05	0.01	<LOD	<LOD	<LOD	<LOD	<LOD	0.03	<LOD	<LOD	<LOD	<LOD	<LOD

[1] Compounds not detected in any samples are not shown.
[2] Values reported below the LOD were replaced with zero to determine Σ_{44}PFAS.
[3] Sum of compounds detected in >95% of samples and included in NHANES 2017-2018 (PFOS, PFHxS, PFNA, PFOA, PFHpS)
[4] **bold** values indicate lab reported values <LOQ,
[5] PFOS is represented as the sum of branched and linear isomers.

Tables S3a-S3c: Unexplained organofluorine (UOF) stratified by sex, race, and age.

Table S3a: Comparing concentration of UOF between male and female donors.

	Unexplained Fluorine (ng F/mL)					
	**N**	**Mean**	**Median**	**SD**	**Minimum**	**Maximum**
Female	10	3.74	3.49	2.08	0.94	7.48
Male	10	3.35	3.18	1.24	1.35	4.98

Table S3b: Comparing concentration of UOF between Black and Hispanic donors.

	Unexplained Fluorine (ng F/mL)					
	**N**	**Mean**	**Median**	**SD**	**Minimum**	**Maximum**
Black	8	3.32	3.12	1.17	1.35	4.98
Hispanic	12	3.70	3.61	1.98	0.94	7.48

Table S3c: Comparing concentration of UOF between donors above and below the median age (48.5).

	Unexplained Fluorine (ng F/mL)					
	**N**	**Mean**	**Median**	**SD**	**Minimum**	**Maximum**
Below Median Age	10	2.83	2.87	1.24	0.94	4.64
Above Median Age	10	4.27	4.35	1.81	1.70	7.48
