# Supplementary material for: Unknown Organofluorine Mixtures in U.S. Adult Serum:Contribution from Pharmaceuticals?
Source: Toxics. 2023 Apr 27;11(5):416. doi: 10.3390/toxics11050416 (PMC10221009; doi:10.3390/toxics11050416)
Supplement: Supplementary file 1 [file toxics-11-00416-s001.zip › toxics-2316311-tex.zip!/Definitions/toxics-2316311-g001.pdf]

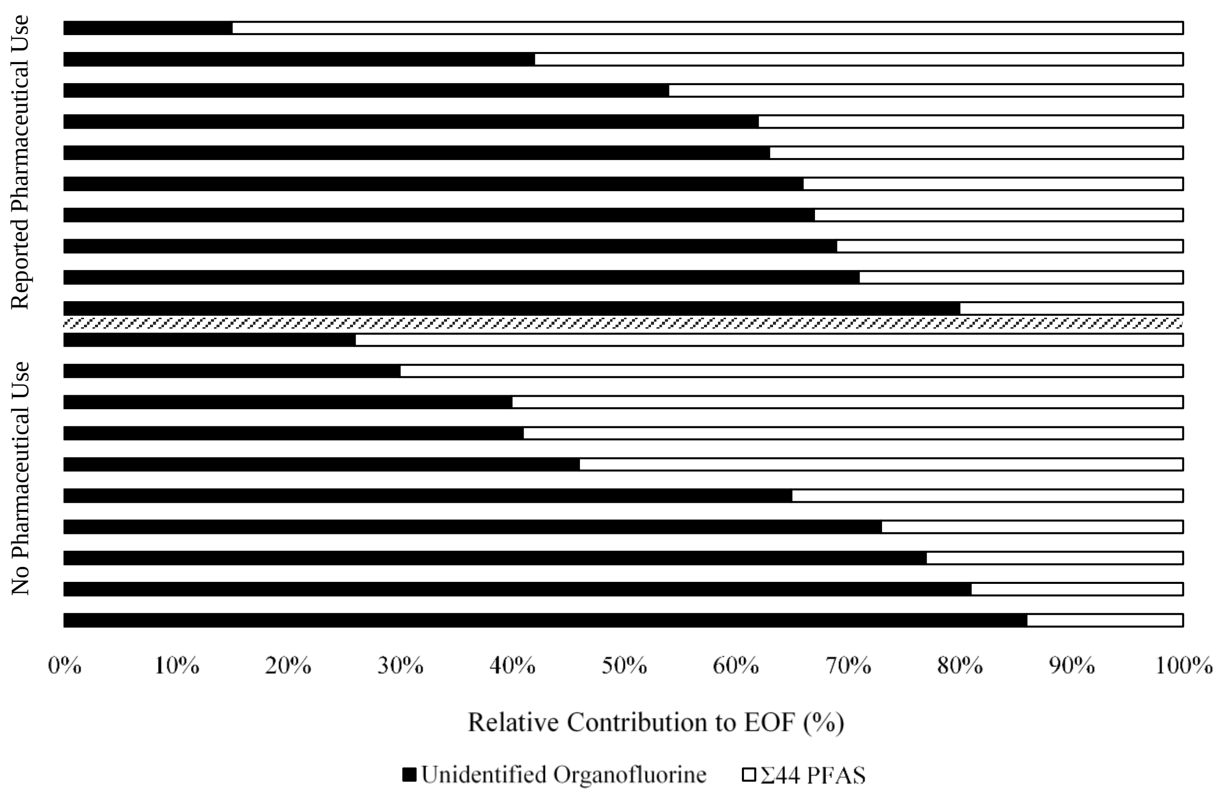

Reported Pharmaceutical Use
No Pharmaceutical Use
0%
10%
20%
30%
40%
50%
60%
70%
80%
90%
100%
Relative Contribution to EOF (%)
Unidentified Organofluorine
Σ44 PFAS
